# Transcriptional suppression of the miR-15/16 family by c-Myc in malignant pleural mesothelioma

**DOI:** 10.18632/oncotarget.27010

**Published:** 2019-06-25

**Authors:** Marissa Williams, Yuen Yee Cheng, Michaela B. Kirschner, Kadir H. Sarun, Karin Schelch, Patrick Winata, Brian McCaughan, Steven Kao, Nico Van Zandwijk, Glen Reid

**Affiliations:** ^1^Asbestos Diseases Research Institute, Sydney, Australia; ^2^Sydney Medical School, The University of Sydney, Sydney, Australia; ^3^PAH Medical Centre, Sydney, Australia; ^4^Chris O'Brien Lifehouse, Sydney, Australia; ^§^Current address: Department of Thoracic Surgery, University Hospital Zurich, Zurich, Switzerland; ^†^Current address: Sydney Local Health District, Concord, Australia; ^#^Current address: Institute of Cancer Research, Department of Medicine I, Medical University Vienna, Vienna, Austria; ^*^Current address: Department of Pathology, University of Otago, Dunedin, New Zealand

**Keywords:** mesothelioma, microRNA, c-MYC, transcriptional repression, tumor suppressor

## Abstract

MicroRNA downregulation is frequent in malignant pleural mesothelioma (MPM), but the mechanisms responsible for loss of miR-15/16 and miR-193a are yet to be elucidated and were investigated in this study. Copy Number Variation (CNV) of microRNA-coding genes was analyzed in MPM cells by digital droplet PCR (ddPCR) and revealed heterozygous loss of miR-193a and miR-15a/16-1, but no change in miR-15b/16-2. Epigenetic control of microRNA expression was inferred following decitabine and Trichostatin A (TSA) treatment which did not substantially affect microRNA expression. Knockdown of c-Myc expression led to upregulation of *SMC4*, miR-15b and 16, and to a lesser extent *DLEU2* and miR-15a, whereas c-Myc overexpression repressed microRNA expression. Chromatin immunoprecipitation (ChIP) assays confirmed the interaction of c-Myc with the *DLEU2 *and* SMC4* promoters. Tumor microRNA expression was determined in samples from MPM patients, with samples of pleura from cardiac surgery patients used as controls. In tumor samples, a strong correlation was observed between the expression of miR-15b and 16 (R^2^=0.793), but not miR-15a and 16. Our data suggest that in MPM, the downregulation of miR-15/16 is due to transcriptional repression by c-Myc, primarily via control of the miR-15b/16-2 locus, while miR-193a-3p loss is due to genomic deletion.

## INTRODUCTION

Malignant pleural mesothelioma (MPM) is a cancer with very poor prognosis and asbestos exposure as the main risk factor, often developing 20-60 years after the date of first asbestos exposure [1]. Median overall survival ranges between 9 and 17 months regardless of stage and the combination of pemetrexed and cisplatin is the current standard of palliative care, offering a modest improvement in patient survival [2]. Attempts to improve disease outcome with targeted therapies were largely unsuccessful [3], highlighting the essential requirement for an improved understanding of MPM biology to facilitate the development of effective therapies. Genetic profiling studies in MPM have identified the upregulation of genes involved in cell-cycle, proliferation and chemoresistance [4, 5] but effective means of attenuating their expression have not yet been determined. More recently targeting immune checkpoints using antibodies specific for the PD-1/PD-L1 axis have produced promising responses in mesothelioma patients [6]. However, compared to the successes observed in melanoma and lung cancer following immune checkpoint blockade [7], disease control is only observed in a fraction of mesothelioma patients in clinical trials with the majority of patients either relapsing or being refractory to treatment [8]. Poor response may be attributed in part to the low mutational load in MPM, which is considered an important determinant for immune checkpoint inhibition response in melanoma and lung cancer [6].

In addition to the changes in the expression of protein coding genes mentioned above, recent evidence implicates noncoding RNAs (ncRNAs) in many of the phenotypes of MPM cells. MicroRNAs (miRNAs) are an important family of short ncRNAs that post-transcriptionally regulate gene expression through interaction with sites in the 3’ untranslated region (3’UTR) of target messenger RNAs (mRNAs). MiRNAs are initially transcribed as long primary microRNA transcripts (pri-miRNAs) by RNA polymerase II that are capped, polyadenylated [9, 10] and subjected to further processing in the nucleus and cytoplasm to generate a mature miRNA [11]. Multiple studies have identified frequent dysregulation of miRNAs in cancer, with many targeting mRNAs involved in tumor progression [12]. The altered expression profiles of miRNAs have assisted in the classification and prognosis of cancer and have been shown to promote tumorigenesis in many malignancies [13] including MPM [14, 15].

As in other malignancies a global downregulation of miRNAs is observed in MPM, with several microRNAs demonstrated to exhibit tumor suppressor activity [16, 17]. MiR-34b/c regulates cell cycle and apoptotic processes via regulation of multiple oncogenic targets including c-Met, CCND1, BCL2, and c-Myc and is downregulated by DNA methylation in approximately 90% of MPM cases [14, 18]. In the case of miR-31, loss is via genomic deletion together with the adjacent *CDKN2A* gene and has been associated with tumor recurrence and aggression in MPM patients [19]. In contrast, the underlying mechanisms responsible for the majority of downregulated miRNAs in MPM are largely unknown. This includes for example, the downregulation of miR-15a/16-1 and miR-15b/16-2 that we recently demonstrated in MPM tumor samples and cell lines. While the anti-tumor functions of miR-15/16 in MPM have been well-characterized *in vitro *and *in vivo *[15] and showed promising response in the phase I clinical trial MesomiR-1 [20], the mechanism(s) leading to their downregulation has not been investigated. Similarly, we have also shown that re-expression miR-193a-3p – another miRNA consistently downregulated in MPM – not only induced apoptosis and reduced cell proliferation of MPM cell lines, but also inhibited MPM xenograft tumor growth to a similar extent as observed with miR-16 [15]. Despite the association of the *MIR193A* gene promoter with CpG islands and its confirmed silencing by methylation in NSCLC [21] and acute myeloid leukemia (AML) [22], methylation was excluded as a predominant cause of its downregulation in MPM cell lines [23] and the causes for its reduction in MPM remain unknown.

In the current study, we aimed to characterize the mechanisms leading to miRNA downregulation in MPM cell lines by systematically determining whether defects occur at different stages of miRNA biogenesis. Results here provide evidence for the contribution of genomic deletion and transcriptional repression – but not epigenetic regulation – to the suppression of mature miR-15a/16-1, miR-15b/-16-2, and miR-193a-3p levels in MPM cell lines.

## RESULTS

### Genomic alterations of miRNA coding genes are observed in MPM

**Figure 1:**
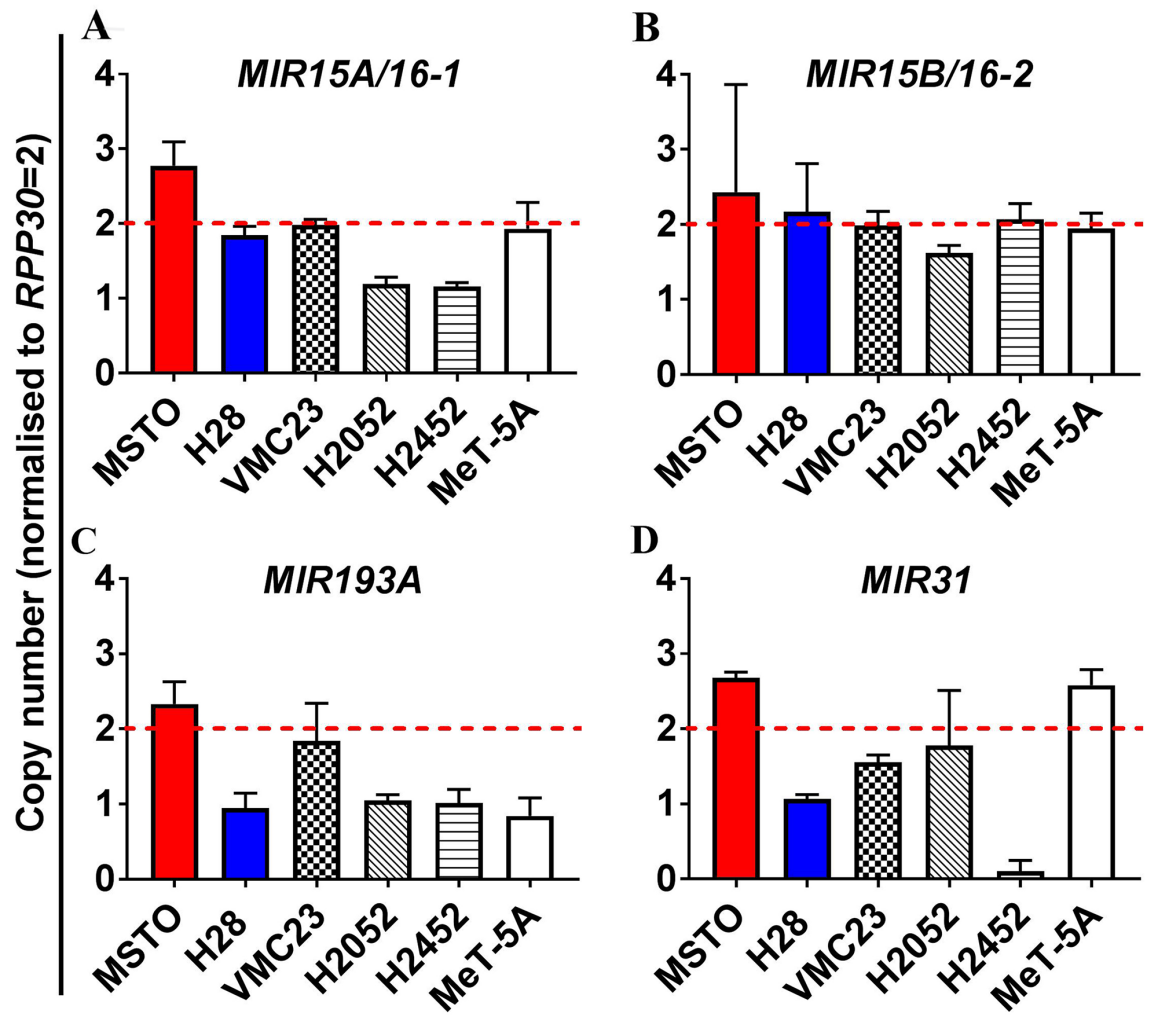
Analysis of copy number variation (CNV) of miRNA genes in MPM reveals allelic loss of the *MIR193A* and to a lesser extent *MIR15A/16-1*, but not *MIR15B/MIR16-2* ddPCR was used to measure the endogenous genomic expression of miRNA coding genes in an MPM cell line panel (MSTO, H28, VMC23, H2052, H2452) as well as the mesothelial cell line MeT-5A. Genomic expression is presented here as copy number determined by normalization to the internal control gene ribonuclease P protein subunit p30 (*RPP30*). Copy number values of <2 were considered to indicate heterozygous deletion while a copy number <1 demonstrated homozygous loss. (A) No genomic loss of *MIR15A/16-1* is determined for MSTO, H28, VMC23 and MeT-5A but heterozygous deletion is observed for H2052 and H2452. (B) No CNV is observed in cell lines for *MIR15B/16-2*. (C) Heterozygous loss of *MIR193A *was observed in H28, H2052, H2452 and MeT-5A. (D) *MIR31* exhibits heterozygous deletion in H28 and homozygous deletion in H2452. *MIR31* loss has previously been identified in H2452 and was included as a positive control for deletion in our cell line panel [19]. All data are the mean of 3 replicate experiments ± SEM.

Copy number variation (CNV) was assessed using digital droplet PCR (ddPCR) in MPM cell lines (Figure 1), revealing heterozygous loss of *MIR15A/MIR16-1* in H2052 and H2452 cells (Figure 1A), but no deletion of *MIR15B/MIR16-2* (Figure 1B). Levels of mature miR-15a are consistently low in the MPM cell line panel [15] regardless of *MIR15A/MIR16-1* CNV (data not shown), suggesting that gene dosage is not solely responsible for the reduced expression of mature miR-15a or miR-16 from the *MIR15A/MIR16-1* locus in MPM. Allelic loss of the *MIR193A* gene was observed in the majority of MPM cell lines tested (Figure 1C), suggesting that heterozygous deletion of the *MIR193A* region is a key contributor to downregulation of miR-193a-3p in MPM.

### Epigenetic changes contribute little to downregulation of miR-15/16 and 193a in MPM cell lines

**Figure 2:**
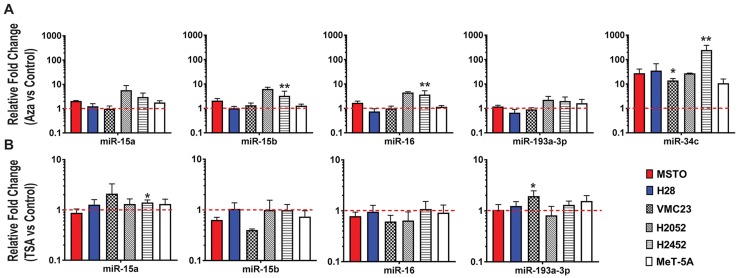
Epigenetic changes do not largely contribute to miR-15/16 downregulation in MPM cell lines **(A)** Expression of miR-15a, 15b, 16, 193a-3p and 34c was determined by RT-qPCR after treatment of MPM cell lines with decitabine (Aza). A significant increase in miR-34c expression was evident following treatment but only subtle changes in expression were determined for miR-15a, miR-15b, miR-16 and miR-193a-3p. **(B)** Changes in miRNA expression after 24 h of treatment with TSA (1 µM) was determined in cell lines by RT-qPCR. MiRNA expression in treated cells was compared to cells treated with a vehicle control and normalized to RNU6B. All data are the mean of 3 replicate experiments ± SEM. * = p ≤ 0.05; ** = p ≤ 0.01.

MiRNA expression was evaluated following treatment with the DNA methylation inhibitor decitabine or the pan-HDAC inhibitor TSA. Decitabine caused a distinct (~250-fold) upregulation of miR-34c (Figure 2A), a miRNA previously shown to be silenced via promoter hypermethylation in MPM [14]. In contrast, negligible changes in expression were observed for miR-15a, miR-15b and miR-16 in most cells, however, the expression of miR-15/16 is partially affected by decitabine treatment in H2052 and H2452, suggesting some regulation by DNA methylation in these cell lines (Figure 2A), whereas miR-193a-3p expression was only slightly altered. Similarly, TSA treatment did not substantially alter expression of miR-15a, miR-15b, miR-16 or miR-193a-3p (Figure 2B). Collectively, this data suggest that methylation and histone deacetylation are unlikely to be major contributors to the loss of miR-15/16 or miR-193a-3p in MPM cell lines.

### Downregulation of pri-miRNA and host gene expression in MPM cell lines

**Figure 3:**
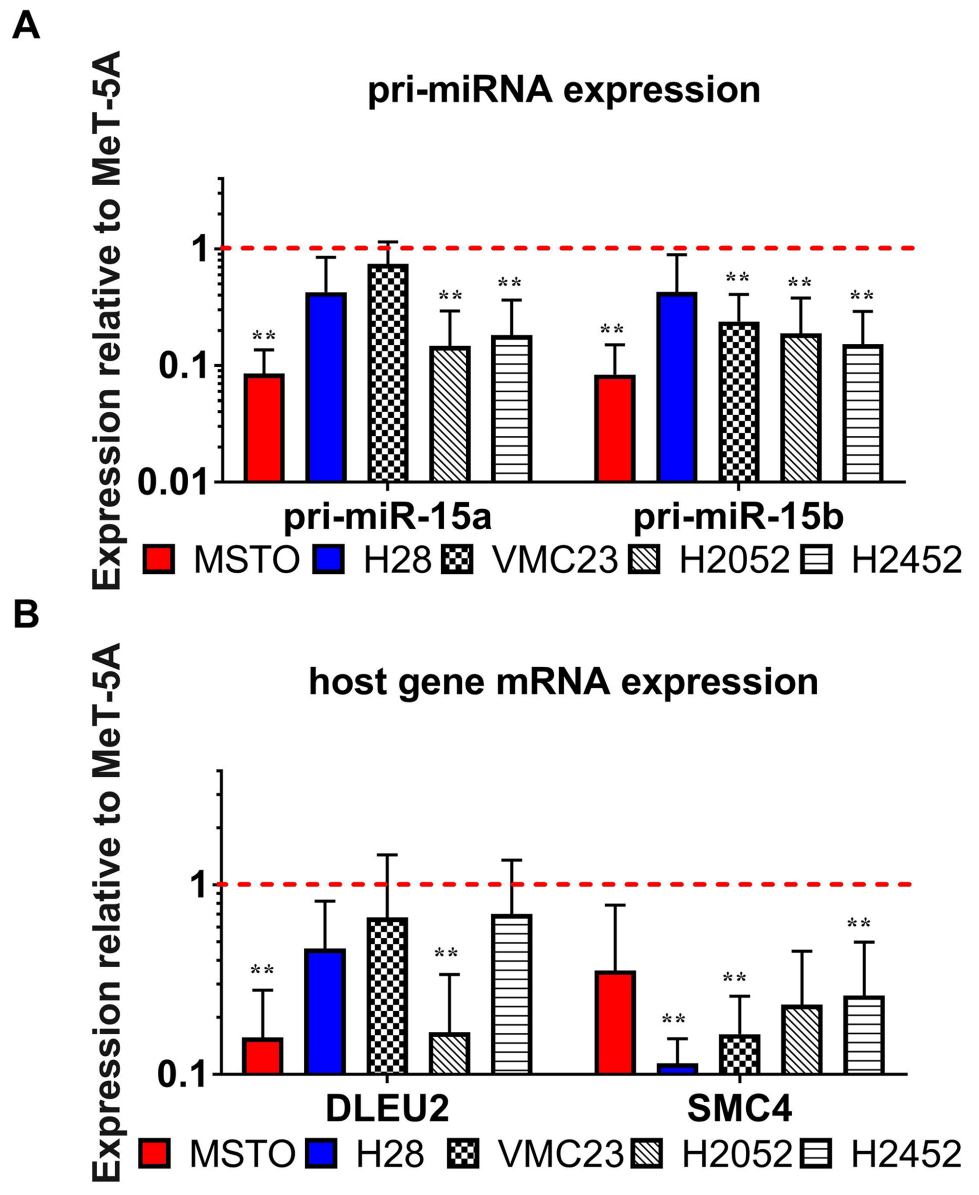
The pattern of expression of miRNA processing intermediates suggest a defect in the transcription of miR-15/16 primary transcripts RT-qPCR was used to test the endogenous expression of miR-15/16 processing intermediates including host gene mRNA/lncRNA and pri-miRNA expression in the MPM cell line panel. **(A)** pri-miR-15a and pri-miR-15b also show downregulated expression in all MPM cell lines compared to MeT-5A (red dotted line). **(B)** host genes *DLEU2* and *SMC4* are transcribed with miR-15a/16-1 and miR-15b/-16-2 respectively and show downregulated expression in MPM cell lines compared to MeT-5A. Expression values were normalized to 18S expression, and are relative to levels in MeT-5A. Results are the mean of 3 biological replicates ± SEM. ** = p ≤ 0.01.

Both pri-miR-15a/16-1 and pri-miR-15b/16-2 were downregulated in most of the MPM cell lines compared to MeT-5A (Figure 3A). Since pri-miR-15a/16-1 and pri-miR-15b/16-2 are transcribed from the promoters of their host genes *DLEU2 *and *SMC4* respectively, we hypothesized that a transcriptional defect at these loci would affect both pri-miRNA and host gene expression. Similar to the observed reduction in pri-miRNA expression, the transcription of DLEU2 – and to a greater extent SMC4 - was downregulated in MPM cell lines compared to the mesothelial cell line (Figure 3B), further supporting a transcriptional mechanism of downregulation.

### Knockdown of c-Myc upregulates miR-15b/16-2 and inhibits cell growth

**Figure 4:**
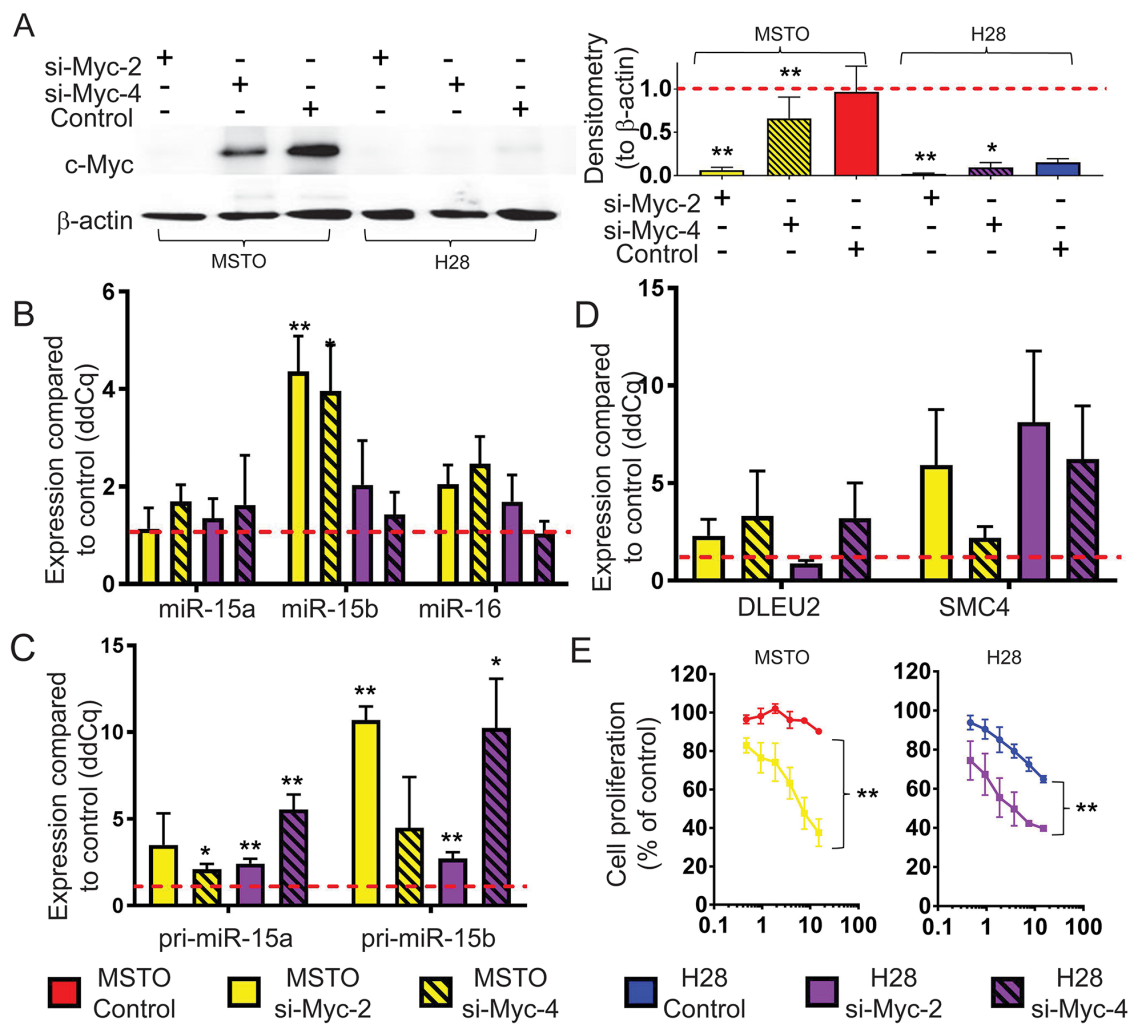
Myc knockdown causes an upregulation of mature miR-15b/16-2 expression and its processing intermediates and inhibits cell growth **(A)** Myc protein expression in MPM cell lines with high (MSTO) and low (H28) basal expression 48 h post transfection with Myc siRNAs (si-Myc-2 and si-Myc-4) and control (all at 20 nM). β-actin expression was included as a loading control. Densitometry was used to quantify Myc protein expression from 3 independent experiments that were normalized to β-actin. Data are mean ±SD. RT-qPCR was used to measure **(B)** Mature miRNA, **(C)** pri-miRNA and **(D)** host gene mRNA expression levels following transfection with Myc siRNA (si-Myc-2, si-Myc-4) or control transfection. RNU6B was used as a reference gene for miRNA expression while 18s was used as reference for pri-miRNA and host-gene expression. Data is the mean from 3 separate experiments ±SD. (E) Cell proliferation was measured 96 h after transfection with si-Myc-2 or control at the indicated concentrations. Cell proliferation is represented as % of untransfected cells. Data is expressed as the mean ±SEM of 3 independent experiments performed in duplicate. * = p ≤ 0.05; ** = p ≤ 0.01.

We next investigated the potential role of the oncogenic transcription factor Myc in miR-15/16 inhibition, as it has been shown to suppress miRNA expression [24-26] and is upregulated in MPM partly due to increased copy number of the *MYC* gene [27]. To determine the effects of Myc on miR-15/16 expression in MPM, we knocked down Myc in MSTO and H28 cells, with high and low Myc expression, respectively. Although basal levels of Myc were substantially higher in MSTO cells, expression in both cell lines was reduced significantly upon siRNA transfection (Figure 4A). Myc knockdown led to upregulation of miR-16 levels in MSTO (2-fold) and H28 (1.7-fold) compared to control transfected cells. In addition, Myc downregulation increased miR-15b (4.4 and 2-fold in MSTO and H28, respectively) but had little effect on miR-15a (Figure 4B). As seen in Figure 4C, levels of pri-miR-15b were increased by 10.7-fold and 2.7-fold in MSTO and H28 respectively following transfection with Myc siRNA while co-transcribed SMC4 was elevated by 5.9-fold in MSTO and 8.1-fold in H28 (Figure 4D). Conversely, levels of pri-miR-15a and DLEU2 showed a more modest increase in expression suggesting that Myc predominantly regulates the miR-15b/16-2 transcript in MPM. As Myc knockdown increased miR-15/16 expression, and these miRNAs inhibit MPM growth *in vitro* and *in vivo *[15], we tested the effect of Myc knockdown in MPM cell line growth. Consistent with the effects of Myc on miR-15/16 expression, Myc knockdown significantly reduced proliferation of MSTO cells (Figure 4E).

### Overexpression of Myc downregulates miR-15b/16-2 and miR-15a/16-1 expression

**Figure 5:**
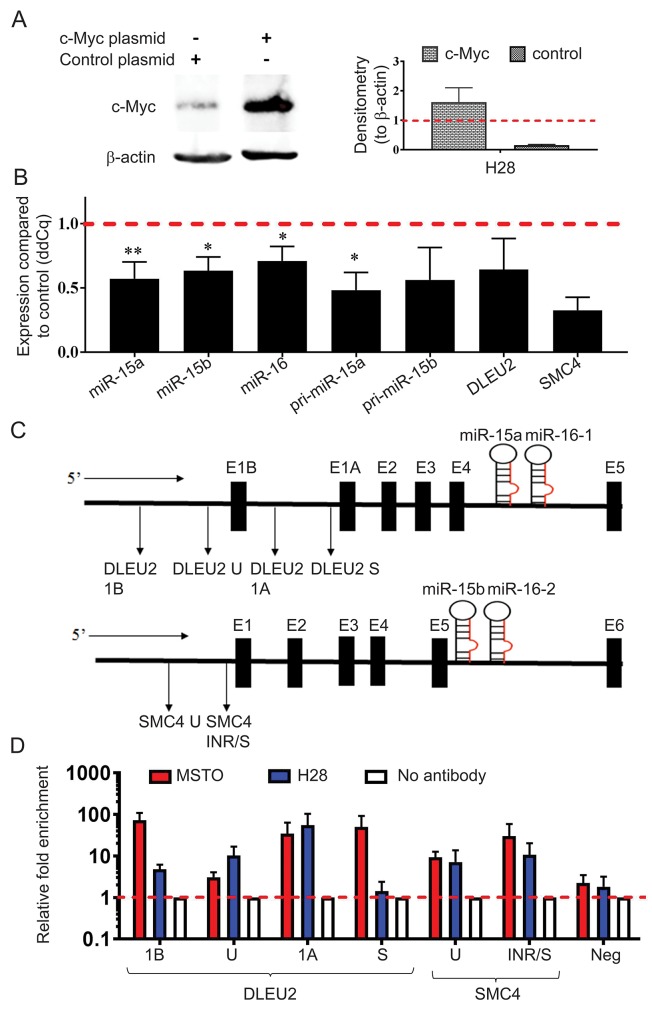
MYC overexpression causes downregulation of miR-15b/16-2 and miR-15a/16-1 and their host genes via binding to the DLEU2 and SMC4 promoters **(A)** H28 cells were transfected with pcDNA3.1(+) and pcDNA3.1(+)MYC plasmids and upregulation of Myc protein expression was confirmed via western blotting with β-actin included as a loading control and normalizer for densitometry. Data are the mean of 3 independent experiments ±SD. **(B)** Mature miRNA, pri-miRNA and host gene expression was analyzed using RT-qPCR with RNU6B and 18S as reference genes. MYC construct transfection induced downregulation of mature, pri- and host gene mRNAs from both the miR-15a/16-1 and miR-15b/16-2 clusters compared to vector control-transfected cells. Data are the mean of 3 independent experiments ±SD. * = p ≤ 0.05; ** = p ≤ 0.01. **(C)** Schematic representation of the promoter regions of the host genes *DLEU2* and *SMC4* with the amplicon locations used for ChIP analysis indicated below each gene. **(D)** ChIP was performed using a c-Myc antibody to detect binding to DNA within the *DLEU2* and *SMC4 *promoter regions. DNA enrichment in chromatin immunoprecipitated by the c-Myc antibody was determined by Real-Time PCR. Relative fold-enrichment was then determined by comparing the enrichment with c-Myc antibodies to the enrichment for the no antibody control (Relative enrichment = sample enrichment (Myc)/sample enrichment (no antibody)). A negative control for Myc binding was included that corresponded to a region in chromosome 1 (chr1:204,366,822-204,366,872). Data are mean ±SEM from 3 independent measurements.

Given the increase of miR-15b/16-2 and miR-15a/16-1 expression following Myc knockdown, we set out to determine the effect of Myc upregulation. Using a MYC expression construct we transiently overexpressed Myc in the low expressing H28 cell line (Figure 5A). Transcription from both the miR-15b/16-2 and miR-15a/16-1 loci were downregulated with Myc overexpression (Figure 5B), consistent with the direct regulation of miR-15/16 by Myc in MPM.

### Myc interacts with the promoter regions of DLEU2 and SMC4 in MPM

Previous studies have demonstrated Myc binding to the promoter region of the *DLEU2* gene that hosts the miR-15a/16-1 cluster [24, 28] but to our knowledge, no previous investigation has linked Myc to the transcriptional repression of *SMC4*, the host gene of the miR-15b/16-2 cluster. To investigate the role of Myc in controlling transcription of miR-15a/16-1 and miR-15b/16-2, we performed chromatin immunoprecipitation (ChIP) experiments and determined enrichment of regions in the *DLEU2* and *SMC4* promoters (shown schematically in Figure 5C). ChIP-qPCR analysis confirmed the association of the Myc protein with the *SMC4* promoter region as seen by 30-fold enrichment of the SMC4 INR/S amplicons in MSTO and 11-fold in H28 (Figure 5D). Similarly, amplicons S, 1A and 1B designed to span the *DLEU2* promoter region produced 50-, 35- and 72-fold enrichment respectively following Myc immunoprecipitation. In amplicons S and 1B enrichment was markedly higher in MSTO samples compared to H28 (Figure 5D), probably due to the higher basal Myc expression in MSTO (Figure 4A). Together, these results support a direct interaction between Myc and both the miR-15a/16-1 and miR-15b/16-2 cluster in MPM.

### The expression of miR-15b and miR-16 is highly correlated in MPM tumor samples

**Figure 6:**
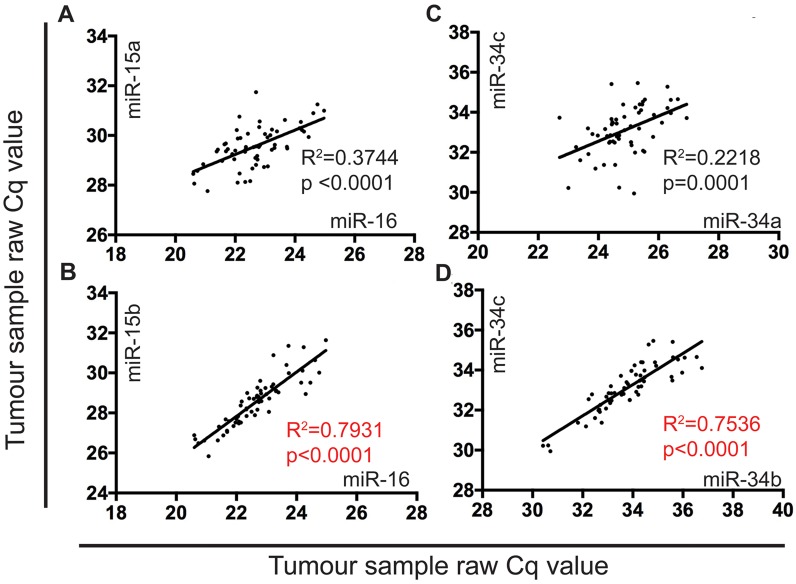
The expression of miR-15b and miR-16 is highly correlated in MPM tumor samples MiRNA expression in FFPE tumor samples (n=60) obtained from patients undergoing EPP were correlated using a linear regression model in Graphpad prism v7. RT-qPCR was used to determine raw Cq values to show correlation of: **(A)** miR-16 and miR-15a (R^2 ^correlation coefficient value of 0.3744), **(B)** miR-16 and miR-15b (R^2^ correlation coefficient value of 0.7931), **(C)** miR-34a and miR-34c (R^2^ correlation coefficient value of 0.2218), and **(D)** miR-34b and miR-34c (R^2^ correlation coefficient value of 0.7536).

Previously we showed that miR-15a, 15b, and 16 were significantly (4 to 22-fold) downregulated in MPM tumors compared with normal pleura [15]. Comparing expression data for each of these miRNAs in individual samples in this data set revealed a strong correlation between miR-15b and 16 (R^2^=0.793) (Figure 6B), but not between miR-15a and miR-16 (Figure 6A). As a comparison, we analyzed expression of the miR-34 family members in the same samples and found that all were downregulated as shown in Supplementary Figure 1 [miR-34a (1.6-fold, P<0.05), 34b (1.8-fold, P<0.01), 34c (2.3-fold, P<0.0001)]. For the miR-34 family, expression of the co-transcribed miR-34b and 34c showed substantial correlation (R^2^=0.753) (Figure 6D), whereas miR-34a and 34b did not (Figure 6C).

## DISCUSSION

Multiple studies have demonstrated global downregulation of miRNA expression in MPM [16], but the underlying mechanisms driving deregulated miRNA expression in MPM remain largely uncharacterized. MiRNAs are frequently located in genomic regions known to be altered in cancer, including minimal regions of loss of heterozygosity (LOH) and amplification as well as fragile sites and breakpoint regions [29]. Copy number alterations in the 13q14 chromosomal region, where miR-15a/16-1 reside, were identified in more than 50% of B-cell chronic lymphocytic leukemia (B-CLL) tumors resulting in loss of mature miRNA expression [30]. In a genome wide study, approximately 25% of both ovarian and breast cancer specimens exhibited copy number loss of regions containing *mir-15a* and *mir-16-1 *[31]. Similarly, the miR-15a/16-1 locus exhibits LOH in NSCLC tumor samples [32] and is frequently deleted in pituitary tumors [33]. Our data are somewhat consistent with these findings; using CNV analysis, we found heterozygous loss of *MIR15A/16-1* in H2452 and H2052 but not in the other MPM cell lines tested. In contrast, no deletions were found in the alternative miR-16 cluster, *MIR15B/16-2* on chromosome 3q25, in any of the MPM cell line panel. Collectively these data suggest that other mechanisms are also accountable for miR-15a/16-1 and miR-15b/16-2 loss in MPM. In comparison, the majority of cell lines were found to have deletion of one allele of the *MIR193A* locus, therefore attributing loss of mature miR-193a-3p expression to genomic deletion of its coding region in MPM.

Epigenetic changes frequently modulate miRNA expression in cancer [34]. Family members miR-34b and miR-34c are inactivated due to CpG methylation in patients with colorectal, pancreatic, mammary, ovarian, urothelial, and renal cell carcinomas and soft tissue sarcomas [35]. In MPM, 85% of tumors exhibited increased methylation of the miR-34b/c promoter [14], and this methylation-induced loss of miR-34c in MPM was confirmed here by the dramatic increases in miR-34c expression in the MPM cell line panel following treatment with the DNMT inhibitor decitabine. In contrast, the hypermethylation of the *MIR193A* gene promoter region found in lung cancer [21], AML [36] and ovarian cancer [37] does not appear to contribute to miR-193a-3p downregulation in MPM, as we have suggested previously [23]. Instead, miR-193a-3p loss in MPM cell lines appears to be the result of genomic deletion. Similarly, expression of miR-15a/16-1 and miR-15b/16-2 changed little in response to DNMT inhibition. These miRNAs were also unaltered in cell lines treated with the HDAC inhibitor TSA. This contrasts with results reported for MCL and CLL, where miR-15a/16-1 transcription was increased by the addition of histone deacetylase inhibitors [38, 39]. Collectively, this data suggests that epigenetic modifications are not a predominant cause of the substantial reduction of miR-15a, 15b and 16 expression in MPM cell lines and tumors.

Having excluded epigenetic modifications as predominant mechanisms causing miRNA loss in MPM cell lines, altered transcription factor activity was examined as a mechanism that could repress miRNA transcription, as found in other cancers [34]. Recently, DNA copy number analysis revealed frequent genomic amplification of the *MYC* gene in MPM tumor samples and cell lines [27]. c-Myc has integral roles in tumorigenesis by controlling genes involved in cell growth [24, 40] and is also implicated in the transcriptional control of miRNA expression [39]. Although Myc is regularly involved in the activation of gene expression, and promotes expression of the oncogenic miR-17-92 cluster in B-cell lymphoma, lung cancer [41] and chronic myeloid leukemia (CML) [42], it has become increasingly clear that Myc directly represses multiple miRNAs in cancer [26, 40, 41, 43, 44]. For example, Myc induced widespread repression of multiple miRNAs in B-cell Lymphoma, including the tumor suppressors miR-15a/16-1, miR-34a and let-7, via direct association with their promoter regions [24]. Myc has been implicated in the transcriptional repression of miR-15a/16-1 expression in B-Cell Lymphoma [24, 39], Ewing’s sarcoma [26] and colorectal carcinoma [25], and was also shown to interact with HDAC3 to indirectly repress expression of miR-15a/16-1 in Mantle Cell Lymphoma (MCL) [39].

In this study we demonstrated Myc mediated repression of both the miR-15a/16-1 and miR-15b/16-2 clusters. Interestingly, Myc reduction led to dramatic increases in expression from the miR-15b/16-2 region compared with more subtle differences seen for miR-15a/16-1. When activating transcription, Myc and its binding partner Max dimerize before binding to gene promoter regions or within the first introns of target gene DNA via canonical DNA binding sites [45]. Alternatively, during transcriptional repression, Myc associates with core promoter regions via protein-protein interactions, where it inhibits positive regulators of transcription [45, 46]. Myc can repress transcription through initiator (Inr) elements in start sites and interaction with promoter-bound transcription factors such as Miz1, Sp1 and Smads [45, 47, 48]. In MPM cell lines, direct association of Myc with the miR-15a/16-1 cluster was demonstrated by enrichment of the *DLEU2* promoter regions as described in previous studies [24-26, 39]. Unlike *DLEU2*, the *SMC4* (miR-15b/16-2) promoter region does not contain predicted Myc binding regions and Myc enrichment was instead identified in regions that are known to harbor Inr sites as examined by promoter regulation prediction software (YAPP, MAPPER2). In addition, data from MPM tumor samples indicated a stronger correlation between miR-15b and miR-16 than between miR-15a and miR-16. As miR-15b is downregulated to a greater extent than miR-15a in MPM tumors [15], and Myc has a greater effect on the miR-15b/16-2 locus, this suggests that the role played by Myc in the reduction of miR-16 in MPM occurs predominantly via suppression of the miR-15b/16-2 locus.

Myc exerts its oncogenic effects by regulating target genes that have roles in multiple processes including apoptosis and cell growth [39], and the dose-dependent reduction in MPM cell line proliferation we observe after Myc knockdown suggests that Myc plays a similar role in MPM cell lines. Previously, we and others have shown that restoring expression levels of the miR-15/16 family results in similar cellular consequences to Myc knockdown [15, 49], so it is likely that increased miR-15/16 expression contributes to the effects on proliferation seen following Myc silencing. In addition to inhibition of MPM cell growth, miR-15/16 suppression may also contribute to other Myc-mediated effects. For example, tumor-specific inactivation of Myc expression was shown to reverse immune evasion by preventing the Myc-induced transcriptional activation of PD-L1 and CD47 [50]. Although Myc was shown to bind the promoters of both genes, it is likely that Myc-induced miRNA suppression also plays a role as miR-15a, 15b and 16 directly regulate PD-L1 expression [51, 52], and furthermore, Myc expression correlates with PD-L1 expression in lung cancer [53]. In this study, patients with tumors positive for both proteins had the poorest prognosis, leading the authors to speculate that Myc expression could serve as a surrogate marker for treatment response. Whether Myc expression influences response to immunotherapy involving checkpoint inhibitors and has value as a biomarker in MPM requires further investigation.

## MATERIALS AND METHODS

### Cell lines and cell culture

The MPM cell lines MSTO-211H, H28, H2052, H2452, and the immortalized mesothelial cell line MeT-5A were purchased from the American Type Culture Collection (Manassas, VA, USA). The VMC23 cell line was a kind gift from Prof Walter Berger (Institute of Cancer Research, Medical University of Vienna, Austria). All cell lines were cultured in RPMI 1640 supplemented with 10% fetal bovine serum (both from Thermo Fisher Scientific, Carlsbad, CA, USA) at 5% CO_2_, 37°C and 95% humidity.

### Patient samples

This study analyzed tumor samples from a previously reported series of MPM patients who underwent extrapleural pneumonectomy (EPP)[15]. This work was approved by the Human Research Ethics Committee (HREC) at Concord Repatriation General Hospital, Sydney (CH62/6/2009/078). Formalin-fixed normal pleural tissue (n=23) was collected from patients undergoing cardiac or aortic surgery as described [15], as part of a study approved by the HREC at RPAH (X10-0342). Written informed consent was obtained from all participants.

### Genomic DNA extraction and CNV analysis using ddPCR

Genomic DNA (gDNA) was collected from MPM cell lines using the QIAamp DNA mini kit (Qiagen, Hilden, Germany) according to manufacturer’s instructions and quantified using a nanophotometer (Implen, Munich, Germany). CNV of the miRNA coding regions was determined by amplifying 40 ng of gDNA with specific primers (35 nM each, listed in Supplementary Table 1) multiplexed with the RPP30 reference gene and Evagreen chemistry using the Bio-Rad QX200™ ddPCR™ system (all Bio-Rad Laboratories, Munich, Germany). (See supplementary information for detailed methods).

### Decitabine (5′Aza-2′deoxycitidine; 5′Aza-CdR) and Trichostatin A (TSA) treatment

MPM cell lines were grown to approximately 25% confluence in 5 cm cell culture dishes and then treated with decitabine (5′Aza-CdR; final concentration 5 μM) or vehicle (equivalent volume; 0.1% DMSO) every 24 h for a total of 120 h of continuous treatment. Treatment with Trichostatin A (TSA) commenced 24 h after cells were seeded at a concentration of 1.5 × 10^5^ in 5 cm cell culture dishes and involved 24 h of exposure to TSA at a final concentration of 1 µM or vehicle (equivalent to 0.05 % DMSO). On completion of drug treatment, changes in miRNA expression were determined by RT-qPCR as described in section 2.5.

### Real-time RT-qPCR

RNA was extracted from MPM cell lines using TRIzol (Thermo Fisher Scientific) as per manufacturer’s instructions, and from laser-capture microdissected FFPE sections as described previously [15]. RNA was quantified using a nanophotometer and stored at -80°C prior to cDNA synthesis. MiRNA was quantified by RT-qPCR as described previously for cell lines and tumor samples [15] using the 2^-ΔΔCq^ method [54] (See supplementary information for detailed methods).

### siRNA transfection

C-Myc and control siRNAs were purchased from GenePharma (Shanghai, China; sequences in Supplementary Table 1). Cells were reverse-transfected with siRNAs using Lipofectamine RNAiMAX (Thermo Fisher Scientific) as described previously [55]. Briefly, siRNAs were applied at a final concentration of 20 nM for expression analysis and at 2-fold serial dilutions starting at 15 nM for determination of cell proliferation. RNA was isolated 24 h and protein 48 h after transfection with siRNAs.

### Western blot

Cells were reverse-transfected with siRNAs in 6-well plates as described above and 48 h later protein was isolated in RIPA buffer and quantified using the Pierce BCA protein assay kit (Thermo Fisher Scientific). Protein expression was measured by SDS-PAGE electrophoresis and Western immunoblotting with a c-Myc antibody (D3N8F; Cell Signaling, Danvers, MA) diluted 1:1000 in blocking buffer overnight at 4°C. Membranes were stripped before probing with a Beta-actin (β-actin) antibody (AC-74, Sigma Aldrich, St. Louis, MO) as a control for protein loading. Chemiluminescence (Clarity Western ECL substrate, Bio-Rad), was used to image bands using the Gel Logic 2200 Imaging system (Kodak, New York, USA). (See supplementary information for detailed methods).

### Generation of a c-Myc expression construct

The coding sequence (CDS) of the c-Myc mRNA (corresponding to the RefSeq entry NM_002467.4) was cloned from total RNA isolated from MSTO cells. The Promega MMLV RT kit was used to reverse transcribe 500 ng total RNA, after which 40 ng of cDNA was amplified using AmpliTaq Gold 360 (Promega) with primers corresponding to the c-Myc CDS (Supplementary Table 1). The resultant PCR amplicon was cloned into the TOPO TA vector (Thermo Fisher Scientific) as outlined by the manufacturer and then sub-cloned into the pcDNA3.1 expression vector (Thermo Fisher Scientific) after sequence confirmation by Sanger sequencing (Ramaciotti Centre, UNSW, Sydney).

### Transient transfections with expression construct plasmids

Plasmids (pcDNA3.1(+)MYC or pcDNA3.1(+)) were introduced into H28 cells by forward transfection with the FuGene transfection reagent (Promega) as per the manufacturer's instructions. RNA was isolated 24 h, and protein 48 h, post transfection. (See supplementary information for detailed methods).

### Chromatin immunoprecipitation (ChIP) qPCR

MSTO and H28 cells were analyzed by ChIP to determine the association of c-Myc with miR-15/16 promoter regions. DNA isolated from untreated cells was subjected to ChIP using the c-Myc antibody with the EZ-ChIP Chromatin Immunoprecipitation Assay kit (Merck-Millipore, Darmstadt, Germany) according to the manufacturer's instructions. qPCR was carried out on the ViiA7 Real Time System with multiple sets of primers specific for the miR-15a/16-1 and miR-15b/16-2 promoter regions as well as a negative control for Myc binding (Supplementary Table 1). Sample enrichment was first evaluated relative to the input (Sample enrichment=2^[Ct input – Ct IP sample]), after which fold-enrichment was calculated by comparing the enrichment with Myc antibodies to the enrichment for the no-antibody control (Relative enrichment = sample enrichment [Myc IP]/sample enrichment [no antibody]). (See supplementary information for detailed methods).

### Cell proliferation assays

The effect of Myc knockdown on the proliferation of MPM cell lines was carried out using SYBR Green-based cell proliferation assays as described [15]. Briefly, cells were seeded at a density of 2.5 × 10^3^ per well in a 96-well plate and reverse-transfected with 30 nM or 60 nM Myc and control siRNAs. After freezing plates at -80°C, proliferation was measured by adding 150 µL of SYBR Green-containing lysis buffer [55] to wells and incubating at 4°C overnight. Plates were equilibrated at room temperature before fluorescence was read on a FLUOstar OPTIMA microplate reader (BMG Labtech, Offenburg, Germany).

### Statistical analysis

Differences in gene expression in cell lines and tumor samples were analyzed with a two-tailed independent samples t-test. Mann-Whitney U test was used to assess differences in cell proliferation. Analyses were performed using SPSS Statistics version 25 (IBM Corp., Armonk, NY) and a *P*-value of ≤0.05 was considered statistically significant. The correlation of tumor miRNA expression was assessed by computing Pearson’s correlation coefficient of linear regression data in Graphpad Prism version 7.0b.

## CONCLUSIONS

In summary, our data provide evidence for the contribution of multiple mechanisms in the downregulation of tumor suppressor miRNAs in MPM. A combination of genomic deletion and transcriptional repression – rather than hypermethylation – contribute to miR-193a, miR-15a/16-1 and miR-15b/16-2 downregulation. Additionally, the mechanisms responsible for suppression of specific miRNAs appear to differ in MPM compared with other malignancies. Interestingly, in MPM, Myc seems to regulate miR-15/16 expression primarily via control of the miR-15b/16-2 locus. This data suggests that regulation of miR-15/16 may be an important contributor to the oncogenic activity of Myc in MPM [25, 26, 39].

## SUPPLEMENTARY MATERIALS



## Abbreviations

MPM: malignant pleural mesothelioma
CNV: copy number variation
ddPCR: droplet digital PCR
TSA: trichostatin A
ChIP: chromatin immunoprecipitation
ncRNAs: noncoding RNAs
miRNAs: microRNAs
3’UTR: 3’ untranslated region
mRNAs: messenger RNAs
pri-miRNAs: primary microRNA transcripts
AML: acute myeloid leukemia
EPP: extrapleural pneumonectomy
HREC: Human Research Ethics Committee
gDNA: genomic DNA
5′Aza-CdR: 5′Aza-2′deoxycitidine
β-actin: beta-actin
CDS: coding sequence
LOH: loss of heterozygosity
B-CLL: B-cell chronic lymphocytic leukemia
CML: chronic myeloid leukemia
MCL: mantle cell lymphoma
Inr: initiator.
