# Supplementary material for: Transcriptional suppression of the miR-15/16 family by c-Myc in malignant pleural mesothelioma
Source: Oncotarget. 2019 Jun 25;10(41):4125–38. doi: 10.18632/oncotarget.27010 (PMC6609241; doi:10.18632/oncotarget.27010)
Supplement: Supplementary file 1 [file oncotarget-10-4125-s001.pdf]

Transcriptional suppression of the miR-15/16 family by c-Myc in malignant pleural mesothelioma

MATERIALS AND METHODS

Genomic DNA extraction and CNV analysis using ddPCR

Genomic DNA (gDNA) was collected from MPM cell lines using the QIAamp DNA mini kit (Qiagen, Hilden, Germany) according to manufacturer's instructions and quantified using a nanophotometer (Implen, Munich, Germany) with readings at 260 and 280 nm. CNV of the miRNA coding regions was determined by amplifying 40 ng of gDNA with forward and reverse primers (35 nM final concentration) for genomic miRNA regions (listed in Supplementary Table 1) and 2X ddPCR Evagreen mastermix (1x) in a 20 µl reaction volume using the Bio-Rad QX200™ ddPCR™ system (all Bio-Rad Laboratories, Munich, Germany). BamHI enzyme (5 units; New England Biolabs) was included in the master mix for the digestion of 40 ng total input gDNA and primers specific for Ribonuclease P/MRP subunit 30 (*RPP30*) (180 nM; Bio-Rad) was included in the multiplex reaction as a reference gene with a copy number of n=2. Droplets were generated using the QX200 Droplet Generator using Evagreen Droplet Generator Oil and the PCR was carried out in a C1000 Touch thermal cycler under the following conditions: 95°C for 5 min, then 40 cycles of 95°C for 30 s, 60°C for 1 min, 4°C for 5 min and 90°C for 5 min after which the reaction was cooled to 12°C for droplet concentration determination. Finally, gene concentrations were read on a QX200 Droplet Reader (all Bio-Rad) as per the manufacturer's guidelines. Data was analyzed using QuantaSoft software (Bio-Rad) where droplet gene population gating was carried out to determine genomic expression and copy number ratios were determined.

RT-qPCR

Analysis of miRNA, pri-miRNA, host genes and mRNA expression was carried out as previously described. Briefly, 100 ng of total RNA was reverse transcribed using microRNA-specific stem-loop primers (Thermo Fisher Scientific, Taqman Assay IDs in Supplementary Table 1) with the MicroRNA Reverse Transcription Kit (Thermo Fisher Scientific) in a 10 µl reaction. The resulting cDNA was amplified by real-time qPCR with microRNA-specific hydrolysis probes (Thermo Fisher Scientific, see Supplementary Table 1 for TaqMan Assay IDs) using the KAPA FAST probe Mastermix (KAPA Biosystems, Wilmington, MA), with reactions carried out in duplicate on the ViiA7 Real Time System (Thermo Fisher Scientific). Each miRNA was normalized to RNU6B and expression relative to controls calculated using the $2^{-\Delta\Delta Cq}$ method [1]. For tumor and pleura samples, RT-qPCR was performed using the TaqMan GeneExpression MasterMix (Thermo Fisher Scientific) according to manufacturer's instructions. Reactions were run in duplicate on an Mx3000P real-time qPCR machine (Stratagene). The miRNA levels in tumor samples were determined using an adapted $2^{-\Delta\Delta Cq}$ method [2] in which the average ΔCq value for all control samples (23 pleura) was used to calculate relative expression in each sample. To determine mRNA, host gene and pri-miRNA expression, total RNA (1 µg) was reverse transcribed using the MMLV reverse transcriptase kit (Promega, Madison, WI) according to manufacturer's instructions. The resultant cDNA was amplified with probe-based assays (Thermo Fisher Scientific; see supplementary Table 1) using the KAPA FAST probe mastermix as described, with expression normalized to 18S using the $2^{-\Delta\Delta Cq}$ method.

siRNA transfection

Myc siRNAs were designed using Thermo Fisher Scientific's Stealth RNAi algorithm to correspond to specific regions within the Myc Open Reading Frame (ORF) region. Myc and control siRNAs were purchased from GenePharma (Shanghai, China) and are listed with their sequences in Supplementary Table 1. siRNAs were reverse-transfected into MPM cells using Lipofectamine RNAiMAX (Thermo Fisher scientific) as described previously [3]. Briefly, siRNAs were applied at a final concentration of 20 nM for expression analysis and 30 or 60nM for determination of cell proliferation, with lipofectamine at a final concentration of 0.8µL/mL; both were diluted in serum free RPMI medium and allowed to incubate for 5 min at room temperature before mixing. Lipofectamine was added drop-wise to the siRNA, and this mixture was left to incubate at room temperature for 20 min to allow formation of the lipoplex. MPM cell lines MSTO and H28 were then added to the siRNA-lipofectamine complex at densities of 1.5×10^5 per well in 6-well plates (for expression) or 2.5×10^3 per well in a 96-well plate (proliferation). RNA was isolated 24 h post-transfection while protein was collected 48 h after transfection with siRNAs.

Western blot

Cells were reverse-transfected with siRNAs in 6-well plates as described above and 48 h later protein was isolated in RIPA buffer (25 mM Tris-HCL (pH 7.6), 150 mM NaCl, 1% (v/v) NP-40, 1% (v/v) Sodium deoxycholate and 0.1% (v/v) SDS) supplemented with protease inhibitors (10mL RIPA buffer + 1X complete mini EDTA-free protease inhibitor cocktail tablets (Roche Diagnostics Australia)) added directly to wells on ice for 30 min. Lysates were scraped from the plate surface and centrifuged at 13 000 rpm for 15 min. Protein from the supernatant fraction was quantified using the Pierce BCA protein assay kit (Thermo Fisher scientific) carried out according to manufacturer's instructions with absorbance measured at 562 nm using the FLUOstar Optima (BMG LabTech, Ortenberg, Germany). Total protein (50 μg) was diluted 4:1 with 1x Laemilli buffer (10% SDS, 50mM Tris-HCL (pH 6.6), 0.002% bromophenol blue) and denatured by boiling for 20 min before separation on a 10% polyacrylamide gel (Separating gel: 10% Stock Acrylamide (29:1), 1x Tris-HCl, 0.1% SDS, 0.075% APS, 0.16% TEMED. Stacking gel: 4% stock Acrylamide, 1x Tris-HCl, pH6.6, 1% SDS, 0.06% APS, 0.46% TEMED) by electrophoresis (100V) with 1X Tris/Glycine/SDS Buffer running buffer (Bio-Rad). Proteins were then transferred overnight at 4°C to a Polyvinylidene Fluoride membrane (Bio-Rad) using the mini trans-blot central core wet system (Bio-Rad) set to low voltage (18 V) with 1X Transfer buffer (3.03 g Tris, 14.4 g Glycine, 200 mL methanol in 1 L MQ water). Membranes were blocked with 5 % w/v BSA (Sigma Aldrich, St. Louis, MO) in TBST (4.85 g Tris, 15.78 g NaCl + 0.1 % Tween 20 in 1 L MQ water) for 1 h at room temperature and incubated with a c-Myc antibody (D3N8F; Cell Signaling, Danvers, MA) diluted 1:1000 in blocking buffer overnight at 4°C. Membranes were washed with 1X TBST and then incubated with a peroxidase-conjugated Anti-Rabbit secondary antibody (ab6721; Abcam, Cambridge, United Kingdom) diluted 1:2500 at room temperature for 1 h. Membranes were stripped using the Restore Western Blot stripping buffer (Thermo Fisher scientific) for 15 min with gentle agitation at room temperature followed by 3X 15 min washes in 1X TBS (4.85g Tris, 15.78g Nacl in 1L MQ water) before probing with a Beta-actin (β-actin) antibody (AC-74, Sigma Aldrich) as a control for equal protein loading. β-actin probing was carried out as described using a peroxidase-conjugated Anti-Mouse secondary antibody (ab6789; Abcam). The Gel Logic 2200 Imaging system (Kodak, New York, USA) was used to obtain protein band images after membrane exposure with chemiluminescent reagent, Clarity Western ECL substrate (Bio-Rad). Quantitative analysis of protein bands was accomplished by densitometry using the Image J 1.48 software. A box of constant size was fit around each band, allowing the density intensities to be calculated by the software. Experimental protein band values were taken as a percentage of values calculated for β-actin and then normalized to control transfected cells.

PCR Cloning

The CDS region of the c-Myc mRNA (corresponding to the RefSeq entry for Homo sapiens v-myc avian myelocytomatosis viral oncogene homologue MYC NM_002467.4) was identified and cloned from total RNA isolated from MSTO cells. The Promega MMLV RT kit was used to reverse transcribe 500 ng total RNA, after which 40 ng of cDNA was amplified using AmpliTaq Gold 360 (Promega) with forward and reverse primers corresponding to the Myc CDS region. HindIII and EcoRI sticky-ends were added to Myc sequences using primers corresponding to the CDS region of Myc, with overhanging corresponding restriction enzyme sequences (see Supplementary Table. 1 for all primers used). The resultant PCR amplicon was incubated with Taq polymerase to add 3' overhangs, then cloned into the TOPO TA vector as outlined by the manufacturer (Thermo Fisher scientific). For cloning into competent DH5-α cells (Thermo Fisher scientific), bacteria were transformed, and single colonies selected on agar plates supplemented with ampicillin (100 μg/mL; Sigma-Aldrich). Plasmid DNA was then isolated using the Qia-prep spin minikit (Qiagen) according to manufacturer's specifications. Inserts were cut by restriction digestion with enzymes HindIII and EcoRI (New England biolabs) and then separated on 1 % agarose gels. Products were excised from the gel and dissolved using the Qia-Quick Gel extraction kit (Qiagen) following the protocol supplied by the manufacturer. Ligation was carried out using T4-Ligation kit (New England Biolabs) to sub-clone the Myc product into the pcDNA3.1 expression vector (Thermo Fisher scientific) after confirmation of sequence identity by Sanger sequencing (Ramaciotti Centre, UNSW, Sydney).

Transient transfections with Expression construct Plasmids

Plasmids (pcDNA3.1(+)MYC or pcDNA3.1(+)) were introduced into H28 (low-Myc expressing) cells by forward transfection with the FuGene transfection reagent (Promega) as per the manufacturer's instructions. Fugene transfection reagent volume was added to 1 μg plasmid at a 3:1 ratio (3 μL) and diluted using serum free RPMI. After 15 min incubation at room temperature this complex was added in a drop-wise manner to 1.2×10^5 cells in 6-well plates. High levels of MYC construct transfection induced cytotoxicity in cells, so cells were instead treated with a pool of 200 ng of pcDNA3.1(+) MYC with 800 ng pcDNA3.1(+) empty vector. Cells were also transfected with 1000 ng of the empty vector pcDNA3.1(+) as negative controls. RNA was collected 24 h post transfection and protein was collected after 48 h.

Chromatin Immunoprecipitation (ChIP) qPCR

MSTO cells have a high basal expression of the Myc protein, while H28 cells have significantly less; for this reason, both cell lines were included in ChIP analysis as models of high and low expressers of the Myc protein, to determine the association of Myc with miR-15/16 promoter regions. Cells were grown in 15 cm tissue culture plates until reaching 80% confluency, after which they were fixed with 1% formaldehyde for 15 min by gentle mixing at room temperature to cross-link proteins and DNA. Cells were washed with cold 1X PBS (Sigma-Aldrich) supplemented with protease inhibitor mixture (1mM phenylmethylsulfonyl fluoride, 1 µg/mL, pepstatin A) after which they were pelleted, lysed, and sonicated to shear chromatin to an average fragment length of 0.2 to 1.0 kb. Resultant DNA fragments were subjected to chromatin immunoprecipitation (ChIP) using the EZ-ChIP Chromatin Immunoprecipitation Assay kit (Merck-Millipore, Darmstadt, Germany) according to the manufacturer's instructions. Briefly, chromatin was pre-cleared by addition of Protein A Agarose/Salmon Sperm DNA (75 µL) for 30 min at 4°C with gentle rotation after which agarose beads were pelleted by brief centrifugation to collect supernatant containing chromatin. DNA was immunoprecipitated with the anti-Myc antibody at a 1:50 dilution overnight at 4°C with gentle rotation, while for a negative control, a no-antibody immunoprecipitation was performed by incubating a sample aliquot with 60 µL of Protein A Agarose/Salmon Sperm DNA. Following Myc antibody incubation, samples were provided with 60 µL of Protein A Agarose/Salmon Sperm DNA and incubated at 4°C for 1 h. Immune complexes from samples and negative controls were then collected by centrifugation and sequentially washed for 5 min with Low Salt, High Salt, and LiCl Salt Immune Complex wash buffers and a TE wash Buffer. Beads were then eluted with 250 µL of elution buffer twice, after which cross-linking of the immunoprecipitated chromatin complexes, no-antibody controls and input controls (10% vol/vol of the total soluble chromatin) was reversed by addition of 5 M NaCl and heating for 4 h at 65°C. Proteinase K (20 mg/mL) was added with buffer to each sample for 1 h at 45°C. DNA was recovered by phenol/chloroform (Sigma-Aldrich) extraction and qPCR was carried out with the multiple sets of primers specific for the miR-15a/16-1 and miR-15b/16-2 promoter regions as well as a negative control for Myc binding (Supplementary Table 1). For the *SMC4* promoter region (Figure 6A) we identified transcriptional activator protein sites known to interact with Myc using transcription factor and core promoter predictor software (MAPPER2, YAPP) and designed real time PCR amplicons for these genomic regions (SMC4 INR/S, SMC4 U- Figure 6A). For the *DLEU2* promoter, we designed real-time PCR amplicons within 4 regions upstream of the transcription start sites of the miR-15a/16-1 cluster (Figure 6A): amplicon U is located directly upstream of the *DLEU2* transcription start site and is also predicted to contain Myc-associated transcriptional activator sites, while amplicon 1A, 1B and S reside within the proximal promoter region and contain binding sites known to be recognised by Myc in literature [4, 5]. Additionally, primers were designed for chr1:204,366,822-204,366,872 as a negative control region for transcription factor binding [4]. Briefly, qPCR involved amplifying 2 µl of Myc immunoprecipitated, no-antibody, and input DNA in a 10 µL reaction containing x1 KAPA SYBR FAST mastermix (KAPA Biosystems) and 2.5 µM forward and reverse primers. The qPCR reaction was then carried out as described on the ViiA7 Real Time System. Sample enrichment was first evaluated relative to the input (Sample enrichment=2^(Ct input – Ct IP sample), after which relative fold-enrichment was calculated by comparing the enrichment with Myc antibodies to the enrichment for the no-antibody control (Relative enrichment= sample enrichment (Myc)/sample enrichment (no antibody)).

Cell proliferation assays

The effect of siRNA induced knockdown of Myc on the proliferation of MPM cell lines was carried out using SYBR Green-based cell proliferation assays as described [2]. Briefly, cells were seeded at a density of 2.5×10^3 per well in a 96-well plate and reverse-transfected with 30 nM or 60 nM Myc and control siRNAs using Lipofectamine RNAiMAX (Thermo Fisher scientific) as described above. Plates were incubated at 37°C for 96 h after which they were frozen at -80°C for a minimum of 2 h to assist with cell lysis. SYBR is a nucleic acid stain that binds to double stranded DNA (dsDNA) to absorb blue light at 497nM and emit fluorescence at 520nM [6]. In this assay, its fluorescence intensity indicates the relative amount of cellular DNA that correlates to the relative number of proliferating cells. Cell proliferation was measured by adding 150 µL of SYBR Green-containing lysis buffer [3] (10 mM Tris/HCl pH=8, 2.5 mM EDTA, 0.1% Triton X-100 with 1:8000 with SYBR green (10000x, Thermo Fisher Scientific)) to wells and incubating at 4°C overnight. Plates were equilibrated at room temperature before fluorescence was read on a FLUOstar OPTIMA microplate reader (BMG Labtech, Offenburg, Germany) set to 485 nm excitation and 535 nm emission.

REFERENCES

1. Livak KJ, Schmittgen TD. Analysis of relative gene expression data using real-time quantitative PCR and the 2(-Delta Delta C(T)) Method. Methods. 2001; 25:402-8. https://doi.org/10.1006/meth.2001.1262. [PubMed]
2. Reid G, Pel ME, Kirschner MB, Cheng YY, Mugridge N, Weiss J, Williams M, Wright C, Edelman JJ, Vallely MP, McCaughan BC, Klebe S, Brahmbhatt H, et al. Restoring expression of miR-16: a novel approach to therapy for malignant pleural mesothelioma. Ann Oncol. 2013; 24:3128-35. https://doi.org/10.1093/annonc/mdt412. [PubMed]
3. Reid G, Wallant NCt, Patel R, Antonic A, Saxon-Aliifaalogo F, Cao H, Webster G, Watson JD. Potent subunit-specific effects on cell growth and drug sensitivity from optimised siRNA-mediated silencing of ribonucleotide reductase. Journal of RNAi and Gene Silencing : An International Journal of RNA and Gene Targeting Research. 2009; 5:321-30. [PubMed]
4. Chang TC, Yu D, Lee YS, Wentzel EA, Arking DE, West KM, Dang CV, Thomas-Tikhonenko A, Mendell JT. Widespread microRNA repression by Myc contributes to tumorigenesis. Nat Genet. 2008; 40:43-50. https://doi.org/10.1038/ng.2007.30. [PubMed]
5. Zhang X, Chen X, Lin J, Lwin T, Wright G, Moscinski LC, Dalton WS, Seto E, Wright K, Sotomayor E, Tao J. Myc represses miR-15a/miR-16-1 expression through recruitment of HDAC3 in mantle cell and other non-Hodgkin B-cell lymphomas. Oncogene. 2012; 31:3002-8. https://doi.org/10.1038/onc.2011.470. [PubMed]
6. Dragan AI, Pavlovic R, McGivney JB, Casas-Finet JR, Bishop ES, Strouse RJ, Schenerman MA, Geddes CD. SYBR Green I: fluorescence properties and interaction with DNA. J Fluoresc. 2012; 22:1189-99. https://doi.org/10.1007/s10895-012-1059-8. [PubMed]

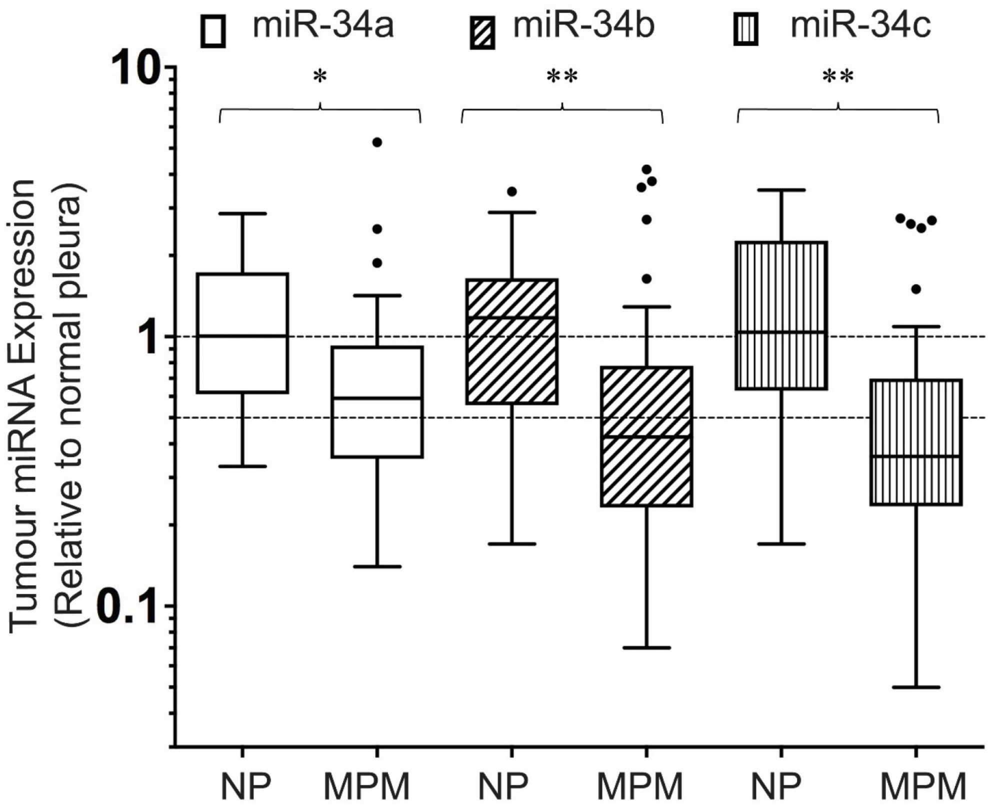

Supplementary Figure 1: The expression of miR-34 family miRNAs are reduced in MPM tumors. Levels of mature miR-34a, miR-34b and miR-34c expression was measured in FFPE tumor samples obtained from patients undergoing EPP (n=60) and related to normal pleura samples (23) using RT-qPCR. Expression was normalized to RNU6B and data was presented as Tukey Box Plot where the median is represented by the solid line within the box and outliers are shown as the dots outside the boxes. * = $p \leq 0.05$; ** = $p \leq 0.01$.

Supplementary Table 1: Assays, primers and siRNA sequences

**TaqMan assays**		
**MiRNA/pri-miR/host gene**	**TaqMan assay ID**	
miR-15a-5p	000389	
miR-16-5p	000391	
miR-15b-5p	000390	
miR-34c-5p	000428	
miR-193a-3p	002250	
RNU6B	001973	
pri-mir-15a	17B04	
pri-miR-15b	17E01	
DLEU2	42A12	
SMC4	42B01	
18s	50A06	
**Primers for dd-PCR**		
**Genomic region**	**Forward primer (5'-3')**	**Reverse primer (5'-3')**
MIR-15A-16-1	ATTCTTTAGGCGCGAATGTGTG	TAC GTGCTGCTAAGGCACTG
MIR-15B-16-2	AGCAGCACATCATGGTTTACAT	CGTGCTGCTAGAGTGGAACA
MIR193A	GATGAGGGTGTCGGATCAAC	CCTCACCACTCCTTCTCCAG
MIR31	GTAATGGGATAAAGGGTGACTGA	GAAGCCTTCTGTGACCATGC
**Primers for ChIP**		
**Genomic region**	**Forward primer (5'-3')**	**Reverse primer (5'-3')**
DLEU2 1B	GCCCCGGGGAGCTGGTTTTC	CCCGCTTCTTCCCCCTCCCA
DLEU2 U	GGCTTTTCTCTCCCAGGA	TGCGTCAACATTGCGAAAA
DLEU2 1A	AAATGCCTGTGGGCTGGTAGCT	GCCGCGGAGGTGAAGTGAACT
DLEU2 S	ATACCGCCTCTTAACCCCCC	CATGCGTAAAAATGTCGGGAA
SMC4 U	CTGGCGAGTCGTTTTTTC	GTGTAGCTTTTGGGATGC
SMC4 INR/S	TGGTCAGGTTGTCACGCT	GCTCAAGGCCTCTATTTTC
negative	AAACCACCCATCCAGAAGGG	CGTGGCAGCACTCGTAAGACT
**Primers for cloning**		
**Insert region**	**Forward primer HindIII (5'-3')**	**Reverse primer EcoRI (5'-3')**
c-Myc CDS mRNA	ATCGAAGCTTATGCCCCTCAACGTTA	ATCGGAATTCTTACGCACAAGAGTTCC
siRNAs	Antisense strand (5'-3')	
Target		
c-81 control	AAGCAACUUGGUAAGACUCGUGUGG	
si-Myc-2	AACAAGUCCUCUUCAGAAAUGAGCU	
si-Myc-4	GGUGAUCCAGACUCUGACC	

MicroRNA, pri-miRNA and host gene assay IDs are listed together with primers for ddPCR, ChIP assay, and cloning and siRNA sequences.
